# Polypoid Adenomyoma of Endocervical Type

**DOI:** 10.1155/2014/275421

**Published:** 2014-07-01

**Authors:** Yuka Takeda, Daiju Araki, Toru Arase, Yutaka Tsutsumi

**Affiliations:** ^1^Fujita Health University School of Medicine, Toyoake, Aichi 470-1192, Japan; ^2^Department of Pathology, Fujita Health University School of Medicine, Toyoake, Aichi 470-1192, Japan; ^3^Department of Obstetrics and Gynecology, Keiyu General Hospital, Yokohama, Kanagawa 220-0012, Japan

## Abstract

We report herein a 53-year-old Japanese female case of polypoid adenomyoma of endocervical type. A sessile 16 mm sized cervical polyp, hard in consistency, was surgically removed. Histologically, the polypoid lesion was composed of smooth muscle bundles and scattered benign-looking endocervical glands. The mucin was diffusely alcianophilic. Immunohistochemically, some mucous glands were positive for MUC1 (CA15-3) and MUC5AC, and the other small glands were immunoreactive for MUC6. MUC2 and mucin characteristic of gastric gland mucous cells (M-GGMC-1 or HIK1083) were negative. Carcinoembryonic antigen was consistently expressed along the apical surface. Estrogen receptor was positive, while progesterone receptor was negative. Ki-67 labeling index was low. These findings were consistent with the endocervical nature of the mucin-producing columnar cells. This is the 18th case of adenomyoma of endocervical type reported in the English literature.

## 1. Introduction

Adenomyoma is a tumor-forming variant of adenomyosis (endometriosis in the myometrium), and adenomyoma is occasionally presented as a cervical polyp [1]. Infrequently, the epithelial component of adenomyoma shows endocervical mucous columnar cells, instead of endometrial glandular cells [2]. A case of polypoid adenomyoma of endocervical type located at the uterine cervix is described here. When the English literature is reviewed [2–9], this should be the 18th case of adenomyoma of endocervical type.

## 2. Case Presentation

A 53-year-old Japanese nulligravida female with a history of simple mastectomy for breast cancer (ductal carcinoma* in situ*) at the age of 45 (with no hormone therapy) received a follow-up health check on June, 2013, and a thumb-sized polypoid lesion was pointed out at the uterine cervix. Five months later, she visited the outpatient clinic of gynecology at Keiyu Hospital, Yokohama, Japan, in order to excise the polyp. Cervical and endometrial cytological examination was negative. She complained of no specific symptoms, including abnormal bleeding. No sexual intercourse was experienced after menopause at 50 years. Imaging disclosed the presence of a 24 mm sized leiomyoma in the otherwise unremarkable (nonenlarged) myometrium. After hospitalization, a sessile 16 mm sized cervical polyp, hard in consistency, located at the direction of one to two o'clock was removed. Postoperative course was unremarkable for 5 months. The patient gave informed consent for reporting the lesion.

**Figure 1:**
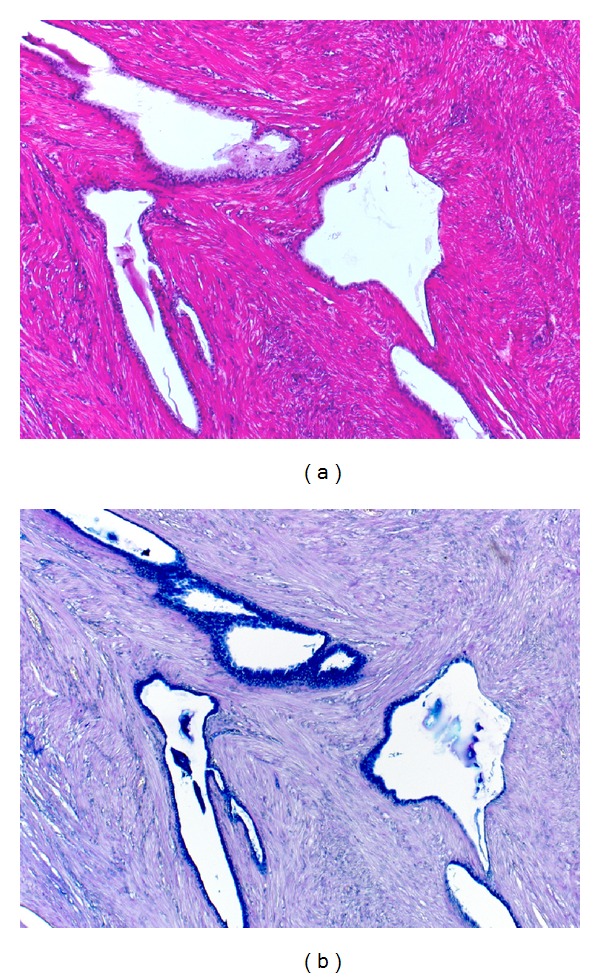
Histologic features of polypoid adenomyoma of endocervical type ((a) hematoxylin and eosin and (b) alcian blue-periodic acid-Schiff). The nodule is histologically composed of benign mucin-producing glandular cells and smooth muscle bundles. The cytoplasmic mucin is solely alcianophilic.

Histologically, the polypoid lesion was lined by compressed endocervical mucosa and composed of smooth muscle bundles and scattered benign-looking endocervical glands. Some glands were dilated, and endocervical mucous cells contained a varied volume of mucin in the cytoplasm. The mucin was diffusely alcianophilic with alcian blue-periodic acid-Schiff reaction, and no magenta-stained neutral mucin was found (Figure 1). Nuclear atypia was hardly observed. No endometrial glandular component was discerned in the lesion. The final diagnosis of polypoid adenomyoma of endocervical type was made.

For characterizing the nature of the glandular component, immunohistochemical study was performed using the amino acid polymer method (Simple Stain Max, Nichirei, Tokyo) after heat-assisted epitope retrieval. Pressure pan heating for 10 minutes was employed. Diaminobenzidine coloring reaction and hematoxylin counterstaining were performed. All mouse monoclonal antibodies (Table 1) were commercially available from Dako (Carpinteria, CA, USA), Novocastra (New Castle, UK), or Kanto Chemical (Tokyo, Japan).

**Figure 2:**
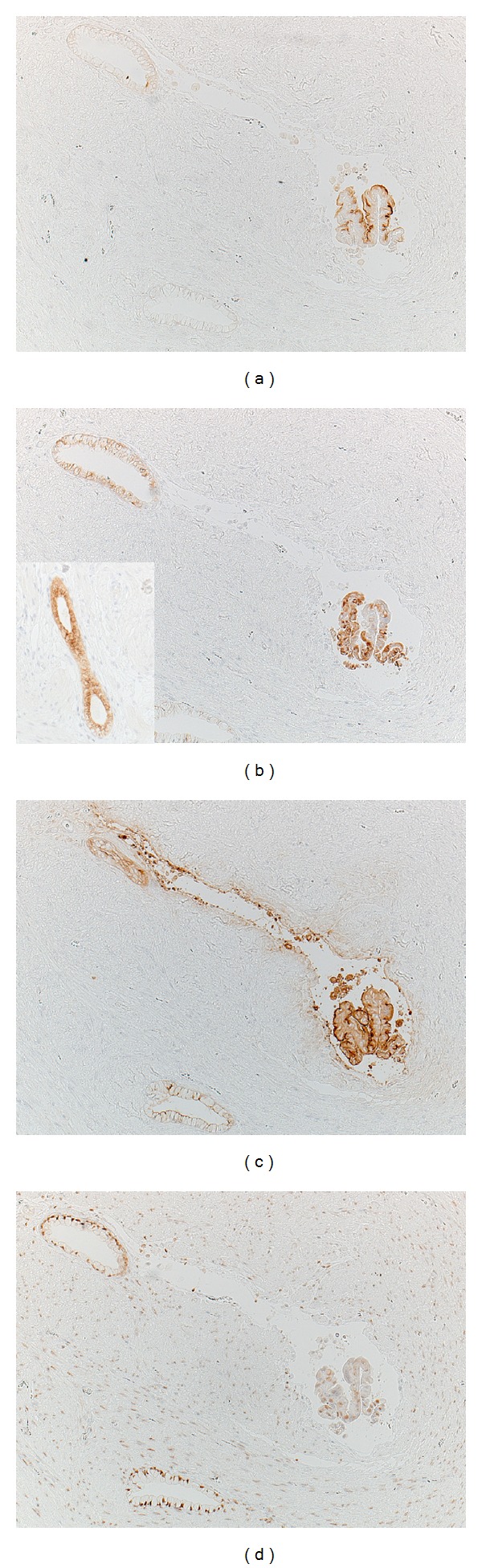
Immunohistochemical features of polypoid adenomyoma of endocervical type using consecutive sections ((a) MUC1, (b) MUC5AC, inset: MUC6, (c) CEA, and (d) ER). The mucin-producing columnar cells express MUC1 (a), MUC5AC (b), and CEA (c). ER is positive in the nuclei of both glandular cells and smooth muscle cells (d). MUC6 reactivity is detected in small glands in other microscopic fields (inset).

Representative immunohistochemical features are demonstrated in Figure 2. The cytoplasm of many mucous glands was positive for MUC5AC, and MUC1 (CA15-3) is expressed along the apical surface of some glandular cells. The other small glands were occasionally immunoreactive for MUC6. MUC2 and mucin characteristic of gastric gland mucous cells (M-GGMC-1 or HIK1083) were negative. Carcinoembryonic antigen (CEA) was consistently expressed along the apical plasma membrane of most glandular cells. Estrogen receptor (ER) was positive in the nuclei of both glands and smooth muscle cells, while progesterone receptor (PgR) was negative. A small number of nuclei of the glands showed nuclear labeling for Ki-67 (5% in hot spots and less than 1% on average). These immunohistochemical findings were consistent with endocervical nature of the mucin-producing columnar cells.

## 3. Discussion

Endocervical nature of the adenomyomatous lesion presented herein was evident, not only by the histological appearance but also by alcianophilia and immunohistochemical positivity of mucin core proteins, CEA, and ER. Expression of mucin core proteins (MUC1 and gastric-type ones such as MUC5AC and MUC6), CEA, and ER in the normal cervical mucous columnar cells has been reported [10–12].

It is well known that adenomyoma of the uterine cervix consisting of endometrial type glands and smooth muscle cells occasionally manifests as cervical polyp [1]. Ten cases of adenomyoma of endocervical type were originally reported by Gilks et al. in 1996 [2], describing that eight of them showed polypoid growth into the endocervical canal. Thereafter, a total of seven case reports appeared in the English literature [3–9]. Of 18 cases of adenomyoma of endocervical type, including the present case, 12 (67%) presented a polypoid mass in the endocervical canal or vagina [2, 4, 6, 8]. One case presented as an endometrial polyp [7]. When the surgical cases were reviewed, there was no description on diffuse distribution of the endocervical glands among the myometrium or “adenomyosis” of endocervical type [2–7].

Particularly, when the adenomyomatous lesion is intramurally located in the uterine cervical wall [2, 5, 9], differential diagnosis from minimal deviation adenocarcinoma of the uterine cervix (adenoma malignum) should be important [2–6, 9]. In the present case, moderate CEA immunoreactivity was observed on the endocervical glandular cells, and Mikami et al. already described CEA immunoreactivity as a diagnostic pitfall in distinguishing from adenoma malignum [4]. CEA positivity was also described by Gilks et al. [2]. Focal expression of MUC6 (gastric pyloric gland mucin core protein) but with negativity for M-GGMC-1 (HIK1083, a carbohydrate antigen on the pyloric gland mucin), as well as the absence of neutral mucin component, was additional histochemical features in the present case. Negative finding for M-GGMC-1 has been emphasized in differential diagnosis between adenomyoma of endocervical type and adenoma malignum [4, 6, 13].

In the present case, the polyp was sessile and hard in consistency, and benign endocervical nature of the glandular component was evident in both histological and immunohistochemical findings. At present, this rare variant of benign adenomyoma of endocervical type is not commented on in most gynecologic pathology textbooks, except for three recently published ones [14–16]. Both pathologists and gynecologists should recognize this rare variant lesion.

## Figures and Tables

**Table 1:** Mouse monoclonal antibodies used in the present study.

Target	Clone	Dilution	Pretreatment	Source
CEA	II-7	1 : 100	EDTA-HIER	Dako
ER	6F11	1 : 50	EDTA-HIER	Novocastra
PgR	16	1 : 100	EDTA-HIER	Novocastra
MUC1	DF3	1 : 500	EDTA-HIER	Dako
MUC2	Ccp58	1 : 500	EDTA-HIER	Novocastra
MUC5AC	CLH2	1 : 200	EDTA-HIER	Novocastra
MUC6	CLH5	1 : 200	EDTE-HIER	Novocastra
M-GGMC-1	HIK1083	1 : 100	None	Kanto Chemical
Ki-67	MIB-1	1 : 100	EDTA-HIER	Dako

EDTA-HIER: heat-induced epitope retrieval in 1 mM ethylenediamine tetraacetic acid, pH 8.0, for 10 minutes using a pressure pan.
